# Sexual and reproductive health and rights through a screen: on technology and accessibility in the Arab region

**DOI:** 10.1093/oodh/oqae017

**Published:** 2024-06-04

**Authors:** Sohayla El Fakahany, Fayrouz Ibrahim

**Affiliations:** Young Experts: Tech 4 Health (YET4H) Research Fellowship, Transform Health Coalition, Geneva, Switzerland; Faculty of Health Sciences, The American University of Beirut (AUB), PO Box 11-0236, Riad El Solh 1107 2020, Beirut, Lebanon; Founding Member, The East Mediterranean Federation of Sexual Health (EMFeSH), Specialty Clinic Building Maamari St. Hamra Beirut, Lebanon; Young Experts: Tech 4 Health (YET4H) Research Fellowship, Transform Health Coalition, Geneva, Switzerland; Senior Programme Specialist. The Center for Development Services (CDS), 957 Corniche El-Nile St., Misr El-Qadima, Cairo 11632, Egypt

**Keywords:** sexual and reproductive health and rights, social non-movement, sociocultural norms, digital platforms, Arab youth, comprehensive sexuality education

## Abstract

In the Arab region, the availability of sexual and reproductive health and rights (SRHR) resources is hindered by structural sociocultural barriers. In response to these challenges, youth and activists have turned to digital platforms such as websites, blogs and social media accounts to improve access to SRHR information and services. The coalescence of such digital platforms has created a complimentary social space that exists to improve the sought for an equal, accessible and empowering SRHR environment. Drawing from Asef Bayat’s concept of social non-movements, this research analyzes SRHR in repressive and conservative settings within the Arab region. The methodology employed includes a digital ethnography on SRHR-related online platforms and an online anonymous survey with young Arab individuals. Through the analysis, it becomes evident that online mobilization efforts may be framed as social non-movements and the role of such digital platforms is imperative to the improvement of youth’s SRHR. Recommendations for improvement include prioritizing comprehensive sexuality education through reformation of curricula, normalizing SRHR and engaging communities in locally and culturally sensitive discourses, disseminating SRHR through media and outreach programs, enhancing accessibility and support, creating more cohesive networks of support and knowledge, and promoting trusted SRHR services and platforms. The coupling between the online space, which has proven to fill in gaps that exist socially, with the formal healthcare system is bound to produce strides in the advocacy for gender equality and equitable access to healthcare in the region.

## INTRODUCTION

Sexual and reproductive health and rights (SRHR) in the Arab region has been a long withstanding topic of contention within several societies. Due to its association with other facts of society, administering cohesive SRHR systems and resources is hindered through different impediments. These impediments are rooted in cultural, religious, social, political and economic factors. There are certain parallels to be drawn amongst the region, while some countries have had significant strides in the sought for a cohesive SRHR system, the general census found through this research is that the Arab region has a particular lack that is significantly obstructive to general societal development, inclusion and access [1, 2]. Isolating SRHR from the multilayered system that it exists within is reprimanding and not conducive to individuals’ actualization of SRHR. The relegation of this fundamental aspect of health and well-being from general healthcare systems must be reconsidered and reviewed.

One of the fundamental components of SRHR is comprehensive sexuality education (CSE). As noted by the WHO: ‘CSE gives young people accurate, age-appropriate information about sexuality and their sexual and reproductive health, which is critical for their health and survival’ [3]. Access to CSE is imperative for the development of gender equity socially, economically and politically. This consideration is not integrated well within medical and social discourse in the region. Given such limitations on the availability and accessibility of CSE, the sought by beneficiaries and provision of it by service providers takes a different route and shape in the mainstream civic space in the Arab region. Each country in the region and its constituent social actors and activists seeks to abridge such gaps through different ways. This gap is exacerbated by the discrepancy between what is included within conventions and treaties and what is provided for citizens from services and care, and most profoundly the provision of CSE. Some of the conventions and treaties that Arab countries have signed and ratified are the Convention on the Elimination of All Forms of Discrimination Against Women, the Convention on the Rights of the Child and the International Covenant on Economic, Social and Cultural Rights. In addition to these conventions, several states also submit Voluntary National Reviews as follow up to their ratification and signatory status to the aforementioned conventions. These submissions are an act of willingness to cooperate with the United Nations to achieve the Sustainable Development Goals, of which include targets that specifically address SRHR (Goal 3: Good Health and Well-being, Goal 4: Quality Education, Goal 5: Gender Equality, Goal 10: Reduced Inequalities and Goal 16: Peace, Justice and Strong Institutions). With these notable stipulations and efforts to enhance their legal status and ascription to international conventions, many Arab states still have a conservative approach to SRHR. These legal subscriptions still exist in tandem with a backdrop of conservatism and a conservative approach to SRHR. Such conservative approach to SRHR is rooted in sociocultural and religious norms that continue to relegate this topic as a shameful topic of discussion that is coupled with morality, rather than a scientific and medical topic of discussion [4, 5].

Although conservatism and social norms directly impact people’s access to SRHR sources and services, significant change is taking place online. It can be said that this change started slowly taking place during and after the Arab Spring revolutions in the 2010s [6]. A prominent example is the Egyptian case, where changes started taking place during and after the January 2011 revolution. During and post revolution, many organizations and advocates started creating and solidifying their presence via online initiatives and platforms to combat sexual violence and gender inequality [7, 8]. These platforms included websites such as ‘*Shoft Taharosh’* (I have seen harassment) and ‘*Harass Map’.* The purpose of these initiatives was to protect the women in the public sphere of the revolution and protests, mainly Tahrir square in downtown Cairo. Through a series of sexual assault and rape cases that surfaced in 2020, a re-emergence of the feminist movement erupted and more traffic was created by online activists and initiatives which echoed the #MeToo movement and started solidifying the online presence of such feminist and SRHR platforms. In June 2020, this mobilization and ‘movement’ started through highlighting high profile sexual assault and harassment cases. Women began garnering confidence to speak out about their personal experiences and legal, medical and social assistance started being mobilized in the online space. This online space, to a large extent, replaced physical spaces for mobilization. What started as reporting and provision of assistance for such topics, quickly morphed into the creation of safe spaces for discussion around topics and issues relating to SRHR, gender justice, sexuality, sexual health (SH), bodily autonomy and rights, and violence against women. Given the timing of this movement in 2020, it became more accessible for individuals to resort to the online space because of the lockdowns and the COVID-19 pandemic. Several founders of such online platforms voiced that the lockdown was one of the reasons they had enough time to work on pressing social issues they were passionate about [9–11].

The lack of mainstream sources on SRHR in the Arab region is reprimanded by the creation of spaces for advocates, healthcare providers and institutions to create platforms that improve people’s access to SRH information online. These platforms include medical websites, blogs and social media accounts. With that, there are also significant inequalities that have risen due to internet and linguistic accessibilities. These inequalities are exacerbated by the gender gap in internet and technological accessibility as indicated by UN Women in 2021 [12–15]. UN Women has published in 2021 that despite the rapid technological advancements going on in the region, these advancements risk further deepening the gendered digital divide in the region. Given such recent developments, access to digital spaces for women is limited, which further complicates women’s participation as active agents in the online social space [12]. It is evident that these online spaces are efficient in disseminating information; however, the dependency on them to substitute the entirety of functioning public healthcare systems is limiting and may possibly further exacerbate its unequally distributed and inaccessible nature. The quality of the public healthcare system, especially when it comes to youth’s and women’s access to SRHR services and information, is weak and online spaces do not have the capacity to provide services and resources in an accessible way to all members of society [16–19].

The aim of this research is to analyze the active digital gender movements in the Arab region vis a vis theory on social non-movements, and analyze the effect of the increased digital platforms and technology on the dissemination of SRHR-related information online to the youth. The research aims to further analyze the effect and utilization of digital platforms as the ultimate sources for SRHR knowledge and services rather than seeking such knowledge and services from registered public health systems and sources. The relationality between such online platforms and officially registered systems outlines the social and economic divides rooted in the Arab region and the relegation of SRHR within the healthcare system and its gendered implications for the society, economy and polity. Through this paper, we seek to analyze the reasoning behind such digital and gendered divides and present ethnographic findings on the unequal access to SRHR as a medical and social right for all.

## MATERIALS AND METHODS THEORETICAL FRAMEWORKS: CURATION OF AN ARAB IDENTITY AND SOCIAL NON-MOVEMENTS

The theoretical framework for this study is based on Asef Bayat’s notion of social non-movements, Social Identity Theory (SIT) and Self-Categorization Theory (SCT). On one hand, Bayat’s notion of social non-movement explains how non-collective actors come together toward a similar goal and seek to cause change through separate and unassociated efforts (2000, 2002 and 2013). SIT explains how individuals establish or develop a sense of self in relation to the social groups to which they belong [20]. As with all societies, social identity plays a significant role in explaining how social dynamics can be drawn out and contextually for understanding how SRHR is framed in the region. Self-Categorization, an adjunct to SIT, builds upon SIT and focuses on the cognitive processes underlying group behavior. It proposes that individuals categorize themselves as members of social groups, and their behavior is guided by the norms associated with their group membership [21]. These frameworks are imperative to assess how young people in the Arab region construct their social identities and their behavior and attitudes toward SRHR. These varying social, political, economic and legal elements influence how young people shape their identities and the contexts within which they exist. This extends to how attitudes toward SRHR are also understood. The curation and construction of social identities influences the nature of the engagement, response or information that is followed by the respective individual. By combining SIT, SCT and social non-movements, this study aims to provide a comprehensive and holistic understanding of how social identity and collective and non-collective actions interact to influence attitudes and behaviors toward SRHR among young people in the Arab region.

In the Arab context, SIT and SCT offer insights into how individuals engage with cultural and social norms and their influence on attitudes and behaviors related to SRHR. Arab social identity builds on insights from SIT and SCT, providing understanding of how identity is formulated and contextualized. Arab individuals are bound by shared language and sociocultural factors, which makes them navigate a shared complex social identity shaped by prominent religions, and family and community dynamics [22, 23]. Living in Arab states where SRHR discussions are often impeded by conservatism further obstruct healthy and informed experiences [1, 2]. In this context, SRHR platform origins do not exclusively determine their audience, given the region’s overall conservative understanding of SRHR. With most platforms using Arabic as their main language, they are more accessible for Arabic-speaking users in the region. Understanding social identity dynamics can inform culturally sensitive and person-centered interventions that promote positive SRHR-seeking behaviors among Arab youth [20].

That being said, it is important to note that culture is rarely characterized by complete uniformity. On the contrary, its dynamism reflects diversity, pluralism and contradictions. There would not have been any need to assert this fact were it not for the misrepresentation of reality by both Western Orientalists and traditional Arab scholars. Western Orientalists have tended to emphasize the ‘constant’ rather than the ‘changing’ nature of Arab culture. Similarly, traditional Arab scholars have tended to emphasize traditional values rather than focus on an evolving, contemporary Arab culture. The distinctiveness of Arab cultural identity needs to take account of a highly complex human reality that includes language, religion, regional affiliation, tribal allegiances, ethnicity and outside forces [6, 24]. All of these factors are constantly changing and developing, both unifying and dividing Arabs at different crossroads. That being said, these categorizations are important because it is only by examining all the nuances of Arab culture and society that we can form an understanding of the Arab identity in the modern world.

Social non-movements, as per Bayat’s theory, are social groups that engage in collective action and activism outside of traditional social movement frameworks (2013). These groups may not have a clear political affiliation or agenda, but they are usually united around shared grievances and aspirations for social change. Social non-movements can take many forms, including street vendors, informal workers and other marginalized groups that are excluded from formal political processes. Despite being non-collective, social non-movements can be an important force for social change, especially in oppressive countries and under repressive regimes, where formal political institutions are weak, ineffective or non-representative of the general public. Social non-movements can challenge dominant power structures and social norms, and they can provide a space for marginalized groups to voice their concerns and demand greater rights or recognition.

This is particularly pertinent to the Arab region due to political complacency and inertia prevalent in its broader political, social and economic landscape. Social non-movements, shaped by non-collective actors engaging in collective action, advocate for the rights of marginalized groups, including women, youth and the urban poor. These actions, as described by Bayat, diverge significantly from revolutions or revolutionary moments in the Arab context, which are typically recognized as discrete events with clear beginnings, middles and ends. Instead, social non-movements evolve gradually over time, embodying persistent everyday actions. Bayat argues that over the past few decades, a social non-movement has been quietly emerging, characterized by subtle yet meaningful shifts [25–29]. Rather than directly confronting authoritarian regimes, individuals in the Middle East increasingly adopt what Bayat terms ‘social non-movements’, comprising fleeting instances of ordinary yet contentious actions. These actions subtly transform everyday conditions, compelling authoritarian governments to respond despite their efforts to prevent them. Unlike traditional movements, social non-movements lack identifiable bureaucratic organizations that governments can target. Instead, they arise from dispersed individuals and groups united by shared experiences and grievances. This reconfiguration of the formal perception of social movements emphasizes the deconstruction of formal social spaces to accommodate mobilization.

SRHR-centered digital platforms are increasingly prevalent throughout the Arab region and they function as agents of change for adequate SRHR-related knowledge. They also serve as symbolic actions of non-collective actors in people’s daily lives [28, 30]. The majority of these platforms operate across the broader Arab region, catering to diverse audiences, who share similar concerns and needs. They prioritize accessibility by disseminating content in Arabic and translating English sources, ensuring broader reach and engagement regardless of geographical location. Moreover, these platforms typically operate online, employing teams of professionals including researchers, content creators, trainers and social workers from various Arab countries. Their shared Arab identity equips them to provide culturally sensitive content and knowledge tailored to address the region’s lack of SRHR literacy and CSE.

The methodological framework for this research encompasses a qualitative approach along with the theoretical framework to analyze digital actions of social non-collective actors. To gather the data, a digital ethnography and an anonymous survey were conducted. The methodology also integrates desk research by incorporating previous studies on the Arab Spring and the digital sphere’s role in both collective and non-collective action, as well as previously published literature and accounts regarding the impact of technology and the globalization of social media platforms on youth’s access to SRHR information in the Arab region.

### Digital ethnography, digital realms

Digital or online ethnography is explained as ethnographic research that is carried out online. Ethnographic fieldwork entails participant observation where the researcher spends long periods of time with the interlocutors in order to ‘to grasp the native’s point of view, his relation to life, to realize his vision of his world’ [31]. Digital ethnography is explained as both an ethnography carried out using technology and an ethnography that collects data via the online sphere by conducting participant observation of online engagement [32–34]. It involves the use of ethnographic methods such as observation, interviews and participant observation to explore the social practices, interactions and meanings that emerge within digital spaces. Digital ethnography is particularly useful for understanding the complex and dynamic ways in which people use and engage with digital technologies, and how these technologies shape their experiences and social identities.

In the context of this study on youth in the Arab region and their SRHR, digital ethnography is an ideal research tool for exploring how youth use digital platforms to access SRH-related information and services, and how these platforms shape their attitudes and behaviors toward SRH issues. By conducting a digital ethnography of prominent digital platforms used by youth in the Arab region, such as social media platforms and online forums, this study aims to gain a nuanced understanding of the role of digital technologies in shaping SRH-related practices, attitudes and identities among young people in this region.

There are several platforms that use the online space to broadcast news or information on gender and SRHR-related experiences and advice, however, the four platforms included in this research are ones that are most used by the survey participants. It is important to note that these platforms are not safe from censorship and safety consequences. The nature of the work these initiatives invest in, research and work on is controversial in the general Arab conservative perspective. This manifests as direct and indirect censorship and control over the kind of knowledge they produce and the kind of information they disseminate. This censorship may include warnings on the content that they are disseminating or reporting and removal of some content, or personal attacks to the teams working on those platforms. This research focuses on four digital platforms from different parts of the Arab region. The platforms are (i) Love Matters Arabic, (ii) *Montada Al Jensaneya* (Sexuality Forum), (iii) This is Motherbeing and (iv) *Mauj* (Waves).

### Qualitative data collection: surveying youth

The research employed a fully anonymous online survey that features short and long answer formats to gather qualitative insights from participants. The participants were recruited using a snowballing sampling technique by which the survey was disseminated online on different platforms. Full anonymity and using online platforms to gather the data were chosen as methods to encourage engagement with the research while mitigating potential fears or feelings of shame often associated with discussions of SRHR due to prevailing sociocultural norms. By conducting the online survey with 60 Arab young participants (aged 18–30), the research aimed to capture their perspectives, experiences, attitudes and interactions with digital platforms focusing on SRHR. The survey addresses three primary objectives: firstly, to scrutinize the influence of social media platforms on the dissemination of SRHR-related information among youth; secondly, and to evaluate the consequences of sole reliance on online platforms versus public and private healthcare services and institutions. Participants were asked to provide demographic information, including age, nationality, gender and relationship status, followed by inquiries regarding their experiences and perceptions regarding SRHR. Questions explored the openness of SRHR discourse in the Arab region, sources typically consulted for SRHR information, awareness of relevant platforms or websites, the perceived increase in accessibility of SRHR information due to digital platforms, personal benefits derived from such platforms, shifts in perspective on SRHR resulting from online information access and acquired knowledge encompassing various aspects of SRHR. Finally, participants were invited to propose recommendations for enhancing SRHR status in their respective countries. This methodology aimed to comprehensively capture insights into the landscape of SRHR knowledge and services in the Arab world amidst technological advancements.

To analyze the findings, a thematic analysis was employed, including an inductive approach, to derive patterns and themes from the collected data. Despite the fact that this study studies accessibility in the Arab region, the survey participants were predominantly Egyptian. With that, there remains to be a flagrant gap in the provision of services in the region and the thematic analysis that follows relays such regional relationality amongst the different countries. Ethical considerations were important in conducting this qualitative research for security reasons. The survey ensured complete anonymization, with researchers having no access to participants’ personal information for security and safety reasons. This was mitigated through the snowballing technique that we employed, whereby we ensured that the process of dissemination of the survey did not pose any security or personal issues for participants and for the researchers.

## RESULTS 

### Online advocacy

Some of the main platforms utilized in the region are: *Mauj*, *Mother Being*, *Montada Jensaneya* and *Love Matters Arabic*. Each of these platforms works in varying capacities, but they all have an informative, user-centered and user-friendly approach. In the context of the Arab region, the nature of this information disseminated on these platforms has become the locus to the type of information that Arab individuals and Arabic-speaking individuals have found useful and have benefited from in their sought for SRHR services. This information has become integral and more centralized than before. This is not the ultimate means through which individuals gain their knowledge; however, these platforms have filled a wide gap in the SRHR field in the region, substituting that of mainstream media. These platforms, Mauj, Mother Being, Montada Jensaneya and Love Matters Arabic, play a pivotal role in addressing SRHR concerns in the Arab region. They offer a range of services and content tailored to the diverse needs of Arabic-speaking individuals, reflecting the complex sociocultural context of the region. In this region, where discussing SRHR can still be considered taboo, these platforms have bridged the information gap, offering credible and culturally sensitive content. They have become trusted sources for individuals seeking guidance on sensitive issues, contributing to improved health outcomes and fostering a more informed and empowered Arab society. The centralization of such information on these platforms highlights their critical role in advancing SRHR and facilitating open discussions in the Arab world. As they continue to evolve and adapt to changing needs, their impact on promoting SRHR in the region is likely to grow even more significant.

There are multiple platforms that provide varying services, whether online and digital or through on ground activities. *Love Matters Arabic* is a platform under RNW media, which started being active in 2013. The platform operates through a website and social media pages on Instagram and Facebook. Love Matters Arabic works with individuals and with providing training on consent, autonomy and other topics. They also offer a wider or more transparent type of information across their social media, which is not the case for Mother Being for instance. Mother Being was founded in 2020 by an Egyptian doula called Nour Emam, This is Motherbeing is the leading Femtech company in the MENA region. Even though the platform started as an awareness building and raising platform, Emam has since then expanded its scope and capacity of work to extend beyond just its platforms. The main focus of the platform was to empower women to gain knowledge of their own bodies, to inform women about pleasure and autonomy. Another female-centered platform is Mauj. Mauj can be considered to provide solely or most prominently for women in the region. Mauj is initially a website and platform based in Lebanon and focuses on CSE. Mauj does not offer additional courses like Mother Being and caters through its informative platform on all issues related to body confidence, bodily autonomy and centralizing female pleasure. This platform is comparable to Jensaneya which is based in Palestine, but also offers information and knowledge to the entire region. Jensaneya however is not only focused on online information through posts, it also offers training for healthcare and SRHR professionals and conducts workshops for trainers. Each of the platforms serves a unique purpose and they all undoubtedly have become formative of the discourse on SRHR in the region.

Although the quality of the knowledge is rooted in medical and scientific resources, the platforms are keen on reshaping existing social norms and misconceptions rather than disseminating rough and flat scientific information that their users might not engage with. Some of these social norms include reworking understandings of virginity, the physiological importance (and lack thereof) of the hymen, importance of consent and consent within marriage, and there are other platforms that work to shed light on contentious cases such as street harassment, gender-based violence cases. Some of the platforms work to promote awareness and in this case, they substitute the lack of existing formal CSE and there are other social media platforms that work to shed light on legal cases and bring awareness to legal cases and the status of those cases. Activist engagement through the platforms produces local knowledge that is transformed to CSE to the users and the public [35, 36]. Arab feminists and gender advocates have long used the internet to call for change. From ‘Uprising of Women in the Arab World’, ‘Girls’ Revolution’ and ‘Revolution against Patriarchal Society’, social movements have started online to advocate for women and girls rights in their Arab region [37, 38]. The concept of social identity at large, and Arab social identity in specific, is important to analyze here because similarly to these movements, digital platforms occur and are created to cater for people from and around the region and not exclusive to the countries they start and are based in.

The lack of collective action and networking among these platforms is a missed opportunity that undermines their collective impact on reshaping social norms, disseminating CSE and advocating for SRHR in the Arab region. Although each social media platform serves a unique purpose and has made significant contributions to the discourse on SRHR, their isolation from one another and from the civic space in each country limits their potential to effect broader societal change. Collaboration and coordination among these platforms could amplify their efforts in challenging prevailing social norms, dispelling misconceptions and promoting awareness on various SRHR issues.

Pooling resources, sharing knowledge and leveraging each other’s expertise would enable these platforms to establish a more cohesive and powerful network. Such a network would not only enhance the impact of their individual initiatives but also create a stronger, united voice advocating for SRHR in the Arab world. It could facilitate the development of standardized, evidence-based content and approaches, ensuring that SRHR information reaches a wider audience while remaining culturally sensitive. Moreover, a collaborative effort could extend beyond online platforms to include partnerships with local organizations, healthcare providers and educators, fostering a holistic approach to SRHR education and advocacy. Ultimately, by breaking down the barriers between these platforms and working together, they can create a more robust and effective movement for positive change in the Arab region’s SRHR landscape.

### Voices of Youth

The survey included responses from 60 individuals, with 1 participant aged 18–21, 26 participants aged 21–25 and 33 participants aged 26–30. Among them, 36 identified as female, 21 as male and 3 as non-binary. Regarding relationship status, 31 participants were single, 24 were in relationships and 5 were married. While all participants were of Arab nationality, the majority were Egyptian, comprising 51 respondents, with 6 from Lebanon, 2 from Morocco and 1 from Tunisia.

The data were analyzed thematically with an inductive approach, whereby the data collected informed the varying themes used in the analysis. While 95% of survey respondents were Egyptians, there was a unanimous agreement on the lack of open discussion about SRHR in the broader Arab region which set an integral tone for the necessity of conducting such a research study. Most of the youth living in the region experience issues with access to SRHR on varying levels. These variable conditions of accessibility are influenced mainly by class, race, gender, sexuality and socioeconomic status. Around 55 of the 60 participants voiced that the topic of SRHR is studied as a taboo and immoral topic, displacing any discussions around this prominent component of health discussions from most social spaces, such as within the family unit, media, schools, institutions, or even in medical institutions and medical schools in the region. The merge of SRHR with morality and politics has made it ever more difficult for it to be discussed in a safe and healthy manner. The sociocultural taboos and misconceptions that are attached to SH and activities have a direct effect on youth’s access to information and services.

Access to comprehensive SRHR information remains a challenge for youth in the Arab region due to sociocultural taboos and inadequate dissemination of information through traditional channels. While traditional media outlets were deemed superficial and unreliable in addressing SRHR, participants highlighted social media platforms as valuable sources of information. These platforms not only provide alternative sources for education but also challenge misconceptions surrounding SRHR. Participants reported using various digital platforms, including Google, gynecologists’ pages and social media, to access information about their bodies and SRHR. However, reliance on digital platforms has its drawbacks, as noted by participants. One male respondent lamented the prevalence of unreliable sources online, including pornography, which perpetuates unrealistic expectations and fear surrounding sexual experiences and health.

Some of the firsthand accounts by the participants can be summarized through the statement made by one of the Egyptian women who noted that, ‘SRHR is usually not discussed openly because people often think it’s *eib* [disgraceful] to discuss anything sex-related’. Another woman emphasized how misconstruing religious beliefs into cultural taboos exacerbates the issue, leading to shame and misinformation regarding SH.

One of the main ways that religion leads to shamefulness is through the merge between morality as stated above and through religious interpretations. ‘Misconstruing religious beliefs into cultural taboos like premarital sex is *haram* [sinful] turns into sex is bad, turns into talking about sex is bad, turns into an embarrassing thing/not enough resources/comfort discussing these things. Reproductive health can be shameful to discuss if the community thinks a new couple should immediately have a child after marriage and yet for some reason they can’t because they might be made to feel “lesser than” and can affect relationships and self esteem’. One of the ways that SRHR continues to be disregarded and ostracized from the forefront of feminist and medical or health discussions in the region is due to issues surrounding morality and disgracefulness. While those are not founded in any scientific or factual basis, they continue to be justification for why it is shameful and taboo to discuss health related issues in a medical and beneficial manner. Another Egyptian woman outlined that, ‘...silence culture about the issue and cultural norms revolving around the idea that having access to information of this kind means that a woman is “impure” and knowledge in this case is considered of a salacious nature. This also relates to the idea of women carrying the “honor” of male relatives including, fathers, brothers, husbands, and sons’.

Despite these challenges, participants recognized the positive impact of digital platforms in shifting societal perceptions of SRHR. The online feminist movement, in particular, was credited with opening doors for discussions on gender-based violence and women’s well-being. Participants expressed optimism about the potential of digital platforms to foster acceptance and understanding of SRHR topics, although they acknowledged the need for continued efforts by individuals and governments to prioritize SRHR education and public services.

Participants in the survey exhibited a wide array of interests on digital platforms, spanning from gaining general knowledge about their bodies to disease prevention and consent education. Common topics of inquiry included general knowledge, such as communication with partners, understanding menstrual cycles, exploring contraceptive methods and learning about the anatomy of reproductive organs. Moreover, participants delved into research on bodily autonomy, focusing on consent, boundaries and rights, showcasing their efforts to empower themselves and advocate for their well-being. Additionally, participants explored anatomical information, including anatomical terminology, menstrual tracking, vaginal care and sexual pleasure, with an aim to deepen their understanding of their bodies. Furthermore, concerns about sexually transmitted infections prompted inquiries about symptoms and coping mechanisms, which also reflect a proactive approach to health management.

When questioned about any shifts in the perception and treatment of SRHR due to digital platforms’ efforts, most participants acknowledged a notable increase in acceptance of the topic. They pointed out that digital platforms, particularly those operated by individuals from the region, have played a crucial role in this change. These platforms not only provide information but also contextualize it to counter prevailing societal taboos and promote open dialog. Despite these advancements, participants emphasized the ongoing need for collective action, urging both individuals and governments to further educate the public on SRHR and offer essential services to address related issues. These findings underscore the importance of accessible and accurate information on digital platforms in promoting SRHR literacy and empowering individuals to make informed decisions about their well-being.

## DISCUSSION

The findings highlight the transformative potential of digital platforms in reshaping societal norms and combating the prevailing discourse of shame and taboo surrounding SRHR. The platforms examined in the research, such as Love Matters Arabic, Montada Al Jensaneya, This is Motherbeing and Mauj, were found to go beyond disseminating scientific information. They actively engage with users, challenge social norms and provide CSE that caters to the unique needs and experiences of Arab youth. The role of media extends and influences the narrative around SRHR extensively in the region; from the formations of social identity to the influence on the type of knowledge that is shared and not censored. In order to promote awareness and normalize SRHR, this must be done through media, including different forms of media. The importance of relying on different media for this is because of the classed and gendered divide in Egypt, whereby internet accessibility is not equal and there are areas with limited to no access to the internet. This can be done through reliance on mainstream media such as television, flyers, door-to-door advertisements. Awareness and information must be provided in local languages as well as in sensitized ways to invite conversations and varying backgrounds of individuals to benefit from them. With that, online platforms must work further at promoting their media and research to their wider audiences, ensuring accessibility in designs, diction and outlook. Expanding their reach is also a positive attribute that such platforms can work toward. In doing so, they can promote clinics, service providers, health professionals and pharmacies through their platforms.

Accessibility and support to young people are detrimental to the advancement of the SRHR field. This accessibility must be promoted through internet accessibility as well as knowledge about all available resources and knowledge. Support can be granted in the form of such access and through the medical professionals that work in this field. Healthcare professionals have a responsibility toward their patients and younger patients to provide the utmost care and attention to their needs and to provide them with the knowledge and information that they need. Furthermore, addressing digital inequalities is essential to ensure that all individuals in the Arab region have equitable access to SRHR information and services. Bridging the digital gender gap, improving internet infrastructure in rural areas, and combating online censorship are crucial steps toward promoting greater inclusivity and advancing SRHR in the region.

One important aspect highlighted in the findings is the role of Arab feminists, gender advocates and social movements in leveraging digital platforms to advocate for women’s and girls’ rights in the region. These movements have laid the foundation for the emergence of digital platforms that address a broader range of SRHR issues beyond sexual violence and harassment. The online sphere has become a space where collective mobilization and activism thrive, promoting social identity and creating an inclusive environment for discussions on SRHR. The research also sheds light on the impact of digital platforms on individual attitudes and behaviors toward SRHR. Participants reported a significant shift in societal acceptance of SRHR topics due to the efforts of these platforms. By contextualizing information and addressing the lack of education and information in conservative societies, the platforms contribute to dismantling the discourse of shame and promoting positive engagement with SRHR. However, participants also acknowledged that there is still work to be done by both individuals and governments to improve education, public services and access to SRHR-related resources.

The findings demonstrate that digital platforms play a crucial role in providing knowledge and information related to SRHR. Participants reported utilizing platforms such as Google and Instagram for various purposes, including understanding bodily autonomy, self-pleasure, menstruation, contraception, sexually transmitted diseases and fertility. The platforms contribute to filling the information gaps and empowering individuals to make informed decisions about their SRHR. Despite the benefits observed, the research highlights certain limitations and inequalities associated with digital interventions. Access to devices and the internet remains a significant barrier, particularly for marginalized groups, exacerbating existing gender disparities in device usage. That being said, while digital platforms are valuable complementary tools, they cannot replace CSE and the guidance of healthcare professionals. Offline interventions, such as reforms in educational systems, family discussions and healthcare support, are crucial for a holistic approach to SRHR. One of the most prominent issues within the education system in Egypt and the region is the fact that it does not adequately address SRHR in medical or scientific curriculum. The suggestion here is to disseminate CSE in schools, which would entail inclusion of SRHR education in school curricula from an early age, normalizing and destigmatizing SRHR education for both genders. The curriculum or teaching criteria for such courses should include teaching concepts of consent, body autonomy and gender equality and should address reproductive problems, infertility and miscarriages in education. This would facilitate conversations amongst youth from a younger age whereby they would also be acquainted with concepts that, if possible, they could discuss with their families. This may lead to a normalization of SRHR within families and within the family structure. This domino effect will fundamentally enable a more cohesive environment for engagement and understanding when it comes to topics such as SRHR.

Despite the ongoing and flagrant support and consumption of social media platforms, there remains to be systematic issues related to accessibility of the internet in the region. Access to the internet in the Arab region has seen significant growth in recent years, with ~66% of individuals and 73% of young people in Arab states using the internet in 2020 [14, 15]. However, this apparent progress masks deeper inequalities that have significant implications for SRHR. One of the most glaring disparities in internet access is the digital gender gap. Women in the Arab region are 12% less likely to use the internet compared to men, making it the largest gender-based digital divide in the world. A staggering 63 million women in the Middle East and North Africa (MENA) region do not have access to mobile internet, which serves as the primary gateway to online content for most people [15]. This gender exclusion can be attributed to various factors, including discriminatory social norms, internet affordability, safety and security concerns, and a lack of digital literacy and skills. In 2020, the exclusion of women from the digital economy resulted in a global GDP loss estimated at $126 billion, underscoring the economic and societal consequences of this digital gender gap. Furthermore, it adversely affects their ability to access crucial SRHR information and services online. Another aspect of internet access inequality in the Arab region is the urban–rural divide. Internet availability, mobile ownership and digital literacy are overwhelmingly in favor of urban areas. According to the International Telecommunication Union, 82% of the urban population in Arab states is covered by a 4G network, compared to only 51% of the rural population. Moreover, urban areas have a higher share of internet users, with 76% of urban residents having internet access compared to only 42% in rural areas [39]. This urban–rural disparity limits access to online SRHR for those in rural regions. Furthermore, censorship and content blockage by internet platforms and other actors exacerbate these inequalities, hindering access to critical information related to SRHR. As a result, individuals in the Arab region, particularly women and those in rural areas, face significant barriers when trying to access comprehensive and holistic SRHR information and services online.

To impose such reformation and development of educational systems, there must be capacity building and development for healthcare professionals, providers and academic personnel. Those responsible for administering such courses and developing courses to be disseminated in educational systems should be those who have received adequate training and are able to disseminate inclusive, sex-positive, and culturally sensitive and relevant information. The approach to such transformation should regard the systematic changes that must be made starting from the individuals responsible for such systems of knowledge to those who are curating this knowledge in a way that can possibly be administered and produce positive impacts at educational institutions.

## CONCLUSION

This research has explored the role of digital platforms in promoting SRHR knowledge and empowerment among youth in the Arab region. The findings demonstrate the transformative potential of these platforms in reshaping societal norms, challenging the discourse of shame and taboo, and providing CSE that caters to the unique needs and experiences of Arab youth. By leveraging digital platforms, Arab feminists, gender advocates and social movements have created inclusive spaces for collective mobilization and activism, promoting social identity and advocating for women’s and girls’ rights. The research highlights the significant shift in societal acceptance of SRHR topics due to the efforts of digital platforms. These platforms have gone beyond disseminating scientific information by engaging with users, challenging social norms and providing a supportive environment for discussions on SRHR. Through contextualizing information and addressing the lack of education and information in conservative societies, digital platforms contribute to dismantling the discourse of shame and promoting positive engagement with SRHR. However, it is important to acknowledge that there is still work to be done by both individuals and governments to improve education, public services and access to SRHR-related resources.

Digital platforms play a crucial role in providing knowledge and information related to SRHR. Participants in the research reported utilizing platforms such as Google, Instagram and dedicated SRHR platforms to gain information about bodily autonomy, self-pleasure, menstruation, contraception, sexually transmitted diseases and fertility. These platforms fill information gaps and empower individuals to make informed decisions about their SRHR. However, it is important to recognize that digital interventions are not a substitute for CSE and the guidance of healthcare professionals. Offline interventions, such as reforms in educational systems, family discussions and healthcare support, are crucial for a holistic approach to SRHR.

Moving forward, it is recommended to focus on CSE that includes SRHR topics in school curricula from an early age, while also addressing reproductive problems, infertility and miscarriages. Current public schooling does not provide extensive knowledge or courses on SRHR, which further creates illiteracy around these topics. Such illiteracy is not only an issue when it comes to the information that youth garner throughout their schooling years but it further creates a gap in the knowledge and in navigating access to services throughout their following youth years. Reforms in educational systems, including the inclusion of SRHR education and capacity building for healthcare providers, are essential. Additionally, awareness-raising campaigns and normalization efforts are crucial in promoting positive attitudes and behaviors related to SRHR. This includes normalizing SRHR through various media platforms, raising awareness in suburban areas and universities, and providing information in local languages to ensure accessibility.

Conclusively, to ensure widespread access to SRHR information and support, collaboration with digital platforms is crucial. Governments and policymakers should recognize the importance of digital platforms in addressing SRHR issues and incorporate them into public health strategies. This includes supporting the efforts of digital platforms in providing accurate and reliable information, collaborating with health professionals and promoting clinics and pharmacies through online platforms. Digital platforms have emerged as powerful tools for promoting SRHR knowledge and empowerment among youth in the Arab region. By leveraging these platforms, challenging social norms and fostering inclusive spaces for discussions, we can contribute to a more informed, empowered and inclusive society that respects and upholds SRHR. 

## Data Availability

The data in this article cannot be shared publicly for the privacy of individuals that participated in the study. The data will be shared on reasonable request to the corresponding author.
